# Diabetes Secondary to Acromegaly: Physiopathology, Clinical Features and Effects of Treatment

**DOI:** 10.3389/fendo.2018.00358

**Published:** 2018-07-06

**Authors:** Francesco Ferraù, Adriana Albani, Alessandro Ciresi, Carla Giordano, Salvatore Cannavò

**Affiliations:** ^1^Department of Human Pathology of Adulthood and Childhood ‘G. Barresi', University of Messina, Messina, Italy; ^2^Department of Clinical and Experimental Medicine, University of Messina, Messina, Italy; ^3^Section of Endocrinology, Diabetology and Metabolism, Biomedical Department of Internal and Specialist Medicine (DIBIMIS), University of Palermo, Palermo, Italy

**Keywords:** acromegaly, diabetes, GH, IGF-1, pituitary tumor, glucose metabolism, impaired glucose tolerance

## Abstract

Acromegaly is a rare disease due to chronic GH excess and to the consequent increase in IGF-1 levels. Both GH and IGF-1 play a role in intermediate metabolism affecting glucose homeostasis. Indeed, chronic GH excess impairs insulin sensitivity, increases gluconeogenesis, reduces the glucose uptake in adipose tissue and muscle and alters pancreatic β cells function. As a consequence, glucose metabolism alterations are a very frequent complication in acromegaly patients, further contributing to the increased cardiovascular risk and mortality. Treatment modalities of acromegaly differently impact on glucose tolerance. Successful surgical treatment of acromegaly ameliorates glucose metabolism abnormalities. Drugs used to treat acromegaly patients may *per se* affect glucose homeostasis, therefore influencing patients' management. Indeed pegvisomant has been shown to positively impact on glucose metabolism, while somatostatin analogs, especially pasireotide, can cause hyperglycaemia. On the other hand, robust data on the effect of dopamine agonists on glycaemic profile are still lacking. This review summarizes the available data on diabetes mellitus in acromegaly patients, with a focus on the potential effects of the medical treatment of the disease on glucose homeostasis, providing an overview of the current state of the art.

## Introduction

Acromegaly is a rare disorder with a reported prevalence of 34–137 cases per million population (1–3). This slowly progressive disease is caused by a chronic excess of growth hormone (GH) and consequently by increased circulating insulin-like growth factor 1 (IGF-1), in most of the cases as a result of a sporadic GH-secreting pituitary tumor (4). Acromegaly is a highly debilitating disease associated with severe comorbidities, including cardiovascular, metabolic, respiratory, neoplastic and musculoskeletal complications, which significantly impact on patients' quality of life and mortality risk (4–7).

GH excess affects insulin sensitivity and gluconeogenesis and can alter pancreatic β-cell function, leading to a derangement of glucose metabolism in a considerable percentage of acromegaly patients (8). Indeed, impaired glucose tolerance (IGT) or diabetes mellitus (DM) are considered a frequent and—in many cases—an early manifestation of acromegaly (7). On the other hand, drugs used to treat acromegaly may cause glucose tolerance abnormalities, regardless of disease control.

This review addresses pathophysiological, epidemiological and clinical peculiarities of DM in acromegaly patients, with a focus on the potential effects of the treatment of the disease on glucose homeostasis.

## Physiopathology

**Figure 1:**
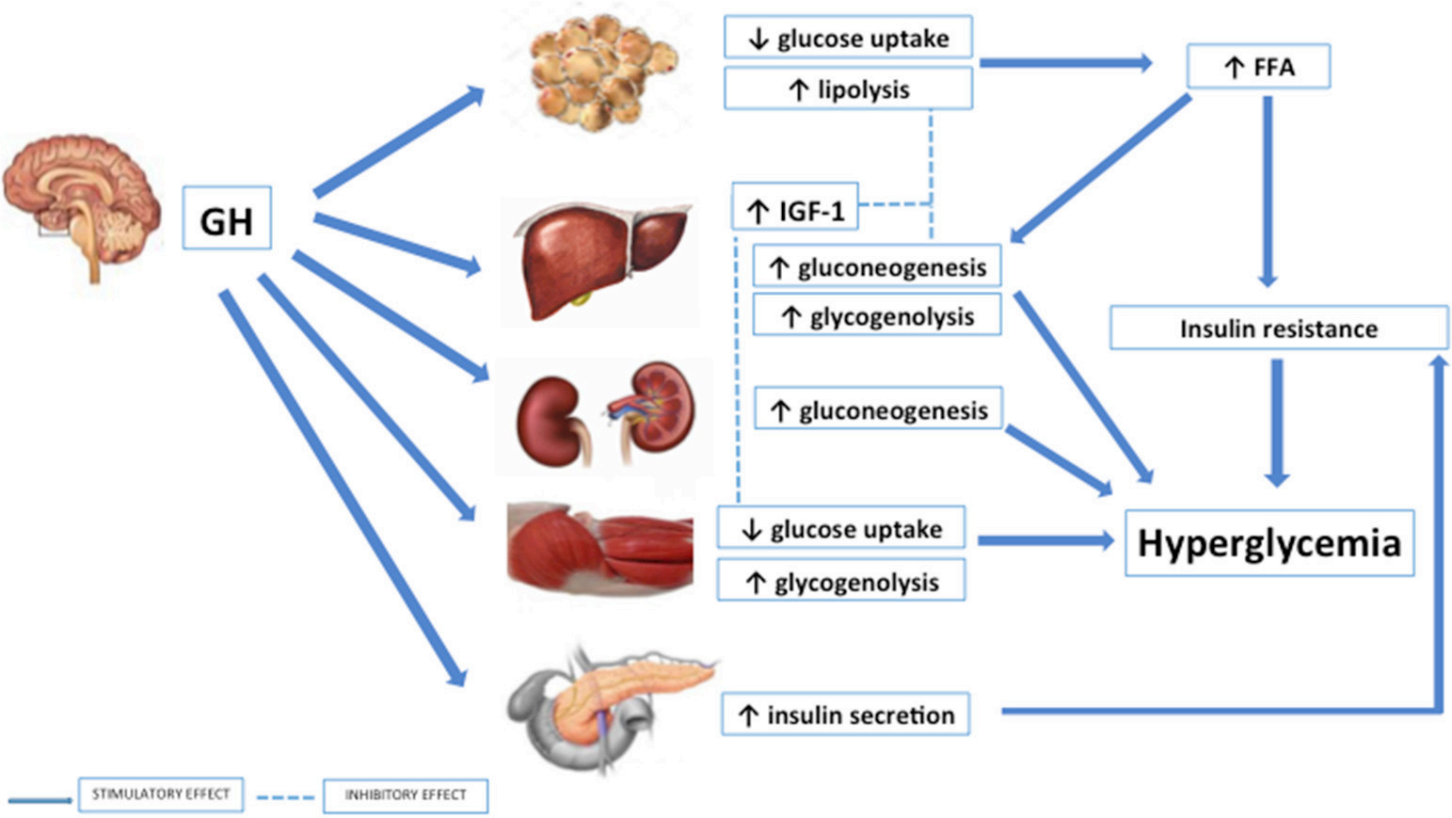
Effects of GH on glucose metabolism.

GH plays physiological effects on glucose metabolism both directly, by inducing gluconeogenesis, glycogenolysis and lipolysis and promoting insulin resistance both in the liver and the periphery, and indirectly, via IGF-1 stimulation, facilitating insulin action. GH inhibits insulin-induced suppression of hepatic gluconeogenesis, thus increasing glucose production. On the other hand, the known lipolytic effects of GH provides free fatty acids (FFA) from the adipose tissue leading to glucose-fatty acid substrate competition and decreased glucose utilization in the muscle (9, 10). The increase in FFA production through the suppression of glucose transporters in adipose tissue cells is one of the factors leading to increased gluconeogenesis and to the development of insulin resistance (11) (Figure 1).

Therefore, the chronic GH excess induces hyperglycaemia by increasing endogenous glucose production and decreasing peripheral glucose disposal in muscle. Conversely, under physiologic conditions IGF-I improves glucose homeostasis and enhances insulin sensitivity primarily on skeletal muscles. Indeed exogenous IGF-1 administration has been shown to promote glucose uptake in peripheral tissues (12) and to reduce serum glucose levels not only in healthy individuals (13) but also in those with severe insulin resistance (14) and type 2 DM (15).

However, in acromegaly patients this effect is not able to counteract the degree of insulin resistance determined by GH excess. A direct GH-mediated alteration of insulin signaling has also been demonstrated. Indeed, GH downregulates the expression of key modulators of insulin signaling and suppresses key signaling pathways involved in stimulation of glucose uptake in muscle and fat, further resulting in insulin resistance (16, 17) (Figure 1). The insulin resistance secondary to GH excess is generally compensated by the increased insulin secretion from the β-cell, while abnormal glucose tolerance may develop when insulin secretion declines. Therefore, the impairment of pancreatic β-cell function with the consequent reduction of insulin secretion significantly contributes to glucose metabolism derangement in insulin-resistant acromegaly patients (8, 18).

The mechanism underlying diabetes in acromegaly are only partially similar to the pathophysiology of classic type 2 DM. Acromegaly patients, in contrast to diabetic non-acromegaly ones, generally have a low amount of visceral fat and insulin resistance is mainly related to GH/IGF-1 excess (9). Indeed, the dysfunction of visceral fat, rather than the total amount, plays a relevant role in determining insulin resistance and impairment of glucose metabolism in patients with acromegaly (19–22). The lipotoxicity in acromegaly may be clinically expressed by the visceral adiposity index, which showed a strong association with the rate of peripheral glucose utilization (*M* value) evaluated through an euglycemic hyperinsulinemic clamp (19). To confirm this, a lipotoxic condition secondary to the lipolytic action of GH has been well described by Freda et al. who showed increased intramuscular adipose tissue, despite a reduction in visceral and subcutaneous adipose tissue, in active acromegaly patients compared with healthy subjects. The increased adipose content in muscle could be associated with GH-induced insulin-resistance (23). Moreover, the adipose tissue dysfunction can cause altered adipokines' secretion that in turn can impact on metabolic profile. In active acromegaly, visfatin has been found to be a reliable index of metabolic alterations, such as insulin resistance and adipose dysfunction (20).

## Epidemiological and clinical features

The disorders of glucose metabolism, including impaired fasting glycaemia (IFG), IGT and DM, are more frequent in patients with acromegaly than in the general population even when considering the groups at higher risk of developing diabetes (24–28). The prevalence of glucose metabolism abnormalities in acromegaly varies significantly among different studies on the basis of study design and population, the setting of patients' evaluation and the adopted criteria for diagnosis and classification of glucose homeostasis alterations. The prevalence of IGT or IFG ranges from 6 to 45% and from 7 to 22%, respectively, while DM has been reported in 16–56% of acromegaly patients (6, 24–35). The prevalence of glucose metabolism alterations has been found to correlate with family history of diabetes, higher BMI and older age as observed in the general population (25, 31). Moreover, in a recent population based cohort study and in the acromegaly Mexican registry, the prevalence or the risk of DM were increased in female patients, although data from other national registries did not show this gender difference (25, 26, 28, 30, 31, 35, 36). However, gender could have a role in determining metabolic alterations, since women with active untreated acromegaly showed higher basal insulin levels and HOMA-IR and lower insulin sensitivity, higher visceral adiposity dysfunction and more frequently features of metabolic syndrome than men, regardless of GH and IGF-1 values (19).

Moreover, acromegaly patients have been reported to develop DM at younger age as compared to general population (25, 31). Furthermore, it has been found that in a variable percentage of patients the diagnosis of DM predates that one of acromegaly, although this could be explained by the known delay in the recognition of this condition (31).

The relationship between GH and IGF-1 levels and glucose metabolism alterations is controversial. In some studies higher GH levels correlated with an increased prevalence of DM, as well as higher IGF-1 levels at diagnosis have been shown to correlate with a higher risk of developing diabetes (24, 25, 30, 31, 36–38). These findings would suggest that the severity of disease could affect the development of glucose tolerance abnormalities as also showed by the increased frequency of DM in uncontrolled patients (39). On the other hand, a longer estimated duration of acromegaly before diagnosis has been also shown to influence the occurrence of glucose intolerance (27, 31).

When considering other GH-excess related comorbidities, hypertension was found to be more frequent in diabetic acromegaly patients and a risk factor for developing DM (31, 36).

In some studies, DM has been found to be a predictor of disease activity, of mortality and of low likelihood of achieving disease control (2, 25). In one retrospective study diabetic acromegaly patients showed higher frequency of malignancies than non-diabetic ones. However, the diabetic patients had higher IGF-1 levels, as compared to non-diabetic acromegalics, that associate *per se* with increased risk of malignancy (40).

Pituitary tumor pathology might correlate with the metabolic status of acromegaly patients since some authors demonstrated that patients with pure somatotroph adenomas were significantly more likely to present with abnormal glucose metabolism than those with mixed adenomas, regardless of GH/IGF-1 levels (41).

Screening of patients with glucose tolerance abnormalities demonstrated that the prevalence of undiagnosed acromegaly in DM patients is 0.6–3%, and 0.13% when including also subjects with IGT (42, 43).

## Effects of surgery and radiotherapy on glucose metabolism

Transsphenoidal (TNS) surgery is the first line therapy in acromegaly. A Polish study involving 239 acromegaly patients documented a significant improvement in glucose homeostasis and insulin sensitivity after TNS surgery. This improvement was not correlated with the achievement of biochemical control (44). Accordingly, in a more recent study, surgical treatment of acromegaly led to an improvement of glucose metabolism either in cured or not cured patients (45). However, once β-cell function is impaired, abnormal glucose metabolism can persist even after surgical cure of acromegaly (46). When surgery is not curative, in selected cases radiotherapy can be used. A retrospective study including 128 acromegaly patients who had undergone radiotherapy showed an improvement of glycaemic profile in diabetic patients. The improvement was correlated with lower GH levels after treatment (47).

## Effects of medical therapy on glucose metabolism

Medical treatment is recommended in acromegaly patients when surgery is not curative or unfeasible. The available medicaments can differently impact on glucose metabolism (8, 48).

### First-generation somatostatin analogs

Octreotide and lanreotide are first-generation somatostatin analogs (SSAs) which display high affinity to somatostatin receptor (sstr) 2 and sstr5 and a weak affinity to sstr3. They represent the first-choice medical therapy for the majority of acromegaly patients.

They can alter glucose homeostasis by reducing pancreatic insulin and glucagon secretion, although the clinical relevance of these effects is controversial. Indeed, it has been suggested that, over the long-term, the decreased insulin secretion could be counterbalanced by the improvement of insulin sensitivity induced by first-generation SSAs (8, 49, 50). On the other hand, although some studies showed an impairment of glycaemic control in acromegaly patients treated with first-generation SSAs, others did not confirm these results (22, 51–53) (Table 1). A meta-analysis of 31 studies performed from 1987 to 2008 showed that first-generation SSAs, used at conventional dosing regimen, do not significantly affect fasting plasma glucose (FPG) levels and do not cause a significant increment in serum glycosylated hemoglobin (HbA1c). Conversely they can lead to a modest impairment of glucose response to oral glucose tolerance test (OGTT) (54). These findings have been confirmed in a multicenter 48-week open label study, using lanreotide autogel as first-line therapy (55). On the other hand, in a recent meta-analysis of 47 prospective interventional studies including 1,297 acromegaly patients, first-generation SSAs were found to affect glycaemic status by reducing insulin, increasing glucose levels after OGTT and HbA1c, with a significant increase of FPG only when they were used as second-line treatment (56).

**Table 1:** Studies on the effects of first-generation SSAs in acromegaly patients.

**First author and year of publication**	**Study design**	**Results**
Giordano et al. (22)	Retrospective, comparative study 12 months follow-up of 231 patients: 151 treated with first-generation SSAs and 80 with surgery as first line therapy	Significant reduction of FPG, HbA1c and DM prevalence in controlled patients in both group Significant reduction in insulinogenic index only in controlled SSAs-treated patients.
Sagvand et al. (59)	Retrospective, comparative, longitudinal case-control study 24 acromegaly patients receiving lanreotide autogel treatment for at least 24 months compared with 39 surgically-cured patients	Mean HbA1c levels similar in both groups. Increased prevalence of DM only in the lanreotide group.
Colao et al. (57)	Open-Prospective study 12 months follow-up of 112 patients receiving primary lanreotide treatment	Glucose homeostasis correlates with the achievement of disease control.
Couture et al. (58)	Retrospective study 42 patients receiving primary lanreotide autogel treatment for a mean period of 23 months.
Valea et al. (53)	Retrospective observational study 12-36 months follow-up of 22 patients treated with lanreotide	
Caron et al. (51)	Retrospective study 12 months follow-up of 25 patients treated with octreotide LAR.	Overall minor clinical impact on glucose mebabolism.
Salvatori et al. (63)	Open-label, multicenter observational study. 24 months follow-up of 241 patients treated with lanreotide autogel.
Mazziotti et al. (54)	Meta-analysis of 31 studies, performed from 1987 to 2008, on acromegaly patients treated with first-generation SSAs for at least 3 weeks	Only modest impairment of glucose response to OGTT
Cozzolino et al. (56)	Meta-analysis of 47 prospective interventional trials treating 1,297 acromegaly patients with first-generation SSAs for at least 6 months	Reduction of insulin levels, increase of after load glucose and of HbA1c levels, without affecting FPG
Mazziotti et al.(61)	*Post-hoc* analysis on 26 patients non-responders to conventional doses of first-generation SSAs and randomized to receive high doses or high frequency octreotide LAR for 6 months	No significant impairment of glucose metabolism with high doses or high frequency lanreotide therapy
Caron et al.(55)	Prospective multicenter open-label single-arm study (PRIMARYS study) 48-week follow-up of 64 patients receiving high doses lanreotide autogel treatment
Caron et al.(62)	Extension of the PRIMARYS study *Post-hoc* metabolic profile analysis
Giustina et al. (60)	Prospective, multicenter, randomized, open-label trial 30 patients partially responders to conventional doses of SSAs randomized to receive high doses or high frequency lanreotide autogel for 24 weeks

*SSAs, somatostatin analogs; FPG, fasting plasma glucose; DM, diabetes mellitus; HbA1c, glycosylated hemoglobin; OGTT, oral glucose tolerance test*.

In a prospective study performed in 112 patients who received first-generation SSAs as first-line therapy, disease control was one of the major predictors of glucose tolerance changes. Indeed, after an increment in FPG levels during the first month of therapy, most of the patients responsive to SSAs treatment experienced no changes or even an improvement in glucose tolerance after 12 months (57). Accordingly, in a retrospective French study, the worsening of glucose homeostasis of acromegaly patients treated with lanreotide was associated with a reduced decrement of GH (58).

In a retrospective longitudinal study, mean HbA1c levels at the last follow-up were not different between patients treated with first-generation SSAs for at least 24 months and only surgically treated cases (HbA1c 6.0 vs. 5.7%, respectively). Nevertheless, the prevalence of DM was increased only in the lanreotide treated group, although both SSAs showed similar effectiveness in terms of disease control (59).

The increase in first-generation SSAs doses or frequency doesn't seem to have a detrimental effect on glucose metabolism. In two different clinical trials, patients non responders or partially responders to standard doses of SSAs were randomized to receive either high dose or high frequency SSAs for 6 months. In both studies, glucose metabolism was not significantly affected by treatment, without any difference between high doses and high frequency (60, 61). High doses treatment has been investigated also in the PRIMARYS study in which patients were treated for 12 months with lanreotide autogel 120 mg/4 weeks. During treatment, glycaemic control improved in patients with DM, as showed by a clinically relevant reduction of HbA1c levels, while no significant changes were observed in non-diabetic patients (62).

Data from the Somatuline Depot for Acromegaly (SODA) registry showed similar mean HbA1c levels after 12 and 24 months of lanreotide treatment in both patients with DM or with normal glucose metabolism. However, after 24 months of therapy, IGF-1 normalization was achieved less frequently in diabetic patients (63).

In conclusion it seems that first-generation SSAs can impact on glucose metabolism. Therefore, periodic glucose monitoring is recommended in these patients.

### Pasireotide

Pasireotide is a multi-receptor ligand SSA which, compared to octreotide, displays a 30-, 5- and 40-times higher binding affinity to sstr1, sstr3, and sstr5, respectively, and a 2.5- times lower affinity to sstr2 (64).

In 2014, two international double-blinded randomized Phase III studies (C2305 and C2402 PAOLA study) showing a superior efficacy of pasireotide compared to first-generation SSAs, allowed to approve it for the treatment of acromegaly patients in whom surgery is ineffective or unfeasible after unsuccessful therapy with first-generation SSAs (65, 66).

In both studies patients treated with pasireotide experienced hyperglycaemia with a higher frequency than those treated with first-generation SSAs. The C2305 was a multicenter randomized 12-month study comparing pasireotide LAR 40 mg/28 days with octreotide LAR 20 mg/28 days. The incidence of hyperglycaemia was higher in patients treated with pasireotide than in those treated with octreotide (57.3 vs. 21.7%, respectively) (65). A crossover extension of this study included 119 patients with uncontrolled disease who have been switched either to pasireotide LAR or to octreotide LAR. Twelve months after crossover, disease control was achieved in 14 and in none of patients treated with pasireotide or octreotide, respectively. Hyperglycaemia occurred in 27.2% of patients treated with pasireotide and in 13.2% of patients treated with octreotide. Discontinuation of therapy because of severe hyperglycaemia was reported in 13.2% of patients treated with pasireotide and in none of the cases treated with octreotide (67).

The multicenter randomized 24-week PAOLA study compared pasireotide LAR 40 and 60 mg with octreotide LAR 30 mg or lanreotide autogel 120 mg in patients with inadequately controlled acromegaly. DM was diagnosed in 21 and 26% of patients treated with pasireotide LAR 40 and 60 mg respectively, whereas no significant changes in glucose metabolism were documented in patients receiving first-generation SSAs (66). Pasireotide effect on glucose homeostasis was similar in both responders and non-responders patients, and glycaemic status before starting pasireotide therapy was predictive of DM development (68). In the ACCESS study, after 3 months of therapy, DM was diagnosed in 40% of patients. A total of 4/42 patients discontinued pasireotide because of severe hyperglycaemia. However, in around 70% of patients, first-line anti-diabetic medicaments were able to manage hyperglycaemia (69).

In the PAPE study, a total of 61 patients with well-controlled acromegaly by means of the combination therapy with first-generation SSAs plus pegvisomant have been switched to pasireotide alone or in combination with pegvisomant. After 24 weeks of therapy, the most frequent adverse event reported was hyperglycaemia (88.5% of patients), with grade 3 and 4 hyperglycaemia occurring in 23% of cases. In the patients who discontinued therapy because of severe hyperglycaemia glucose metabolism fully recovered. The most relevant predictor for development of DM was HbA1c level at baseline (70).

The hyperglycaemic effect of pasireotide can be explained by its affinity binding. Glucagon-producing α cells predominantly express sstr2, whereas insulin-producing β cells mainly express sstr2 and sstr5. Pasireotide, by binding with high affinity sstr5, suppresses insulin secretion, whereas the inhibitory effect on glucagone secretion is only modest. This insulin-glucagon unbalance could explain the overall increase of glucose levels (71). Accordingly, the co-administration of pasireotide and lanreotide in rats do not cause a significant increment in glucose levels. This could be explained by the greater activation of sstr2 by lanreotide, resulting in a balance of the insulin-glucagon ratio (72). Furthermore, pasireotide seems to reduce incretin response, whereas insulin sensitivity is not affected (71). In a 1-week phase I study, performed in healthy volunteers, vildagliptin and liraglutide were more effective than metformin or nateglinide in minimizing pasireotide associated glucose levels alterations, confirming the existence of an abnormal incretin response (73). Indeed, before starting pasireotide therapy patients should undergo an assessment of glucose metabolism and, in diabetic patients, hypoglycaemic treatment should be optimized before starting pasireotide (8).

### Novel somatostatin receptor ligands

New SSAs are being developed/tested aiming to overcome the diabetogenic effects of the actually available molecules. Somatoprim is a compound, not yet commercially available, that binds to human sstr2, sstr4, and sstr5, proven *in vitro* to reduce GH secretion from GH-secreting pituitary tumors and devoid of significant effects on insulin and glucagone secretion (74).

AP102 is a new dual sstr2/sstr5-specific SSA proven to reduce GH levels without causing hyperglycemia during acute or chronic administration in a healthy rat model (75).

### Pegvisomant

Pegvisomant (PEG) is a genetically-engineered GH-analog which, antagonizing GH action at the GH receptor level, leads to lowered IGF-1 production (76). Several studies showed a positive impact of PEG on glucose metabolism, which is actually expected considering the glucose homeostasis changes following GH signaling activation. The effects of long-term treatment with PEG have been assessed in a study including 160 patients in whom fasting serum insulin and glucose levels decreased significantly during PEG treatment, although none of these patients was diabetic at baseline (77). In another study, performed in five patients with acromegaly and insulin-resistance, treatment with pegvisomant for 12 weeks improved metabolic parameters, reducing significantly fasting insulin level, FGP and HbA1c. This decrement was progressive and reached the maximum level after 6 months of treatment (78). When compared to first-generation SSAs, PEG shows a better impact on glucose homeostasis and insulin-sensitivity, as evidenced by studies in patients switched from octreotide to PEG therapy (79, 80). Similarly, co-treatment of acromegaly with first-generation SSAs and PEG has a better effect on glucose profile compared to patients receiving first-generation SSAs only (81). A prospective study showed that FPG and glucose levels after OGTT were lower in patients treated with PEG compared with those treated with first generation SSAs, while insulin resistance improved in all patients with controlled acromegaly (82). In another prospective study, efficacy and safety of PEG treatment have been evaluated in 16 patients resistant to first-generation SSAs. After 12 months of PEG treatment, glucose and insulin levels and HOMA index decreased significantly. Glucose levels improved in all patients with DM or IGT at baseline, even in those who had experienced difficult-to-manage hyperglycaemia during first-generation SSAs treatment (83). While several studies have demonstrated the positive impact of PEG on FPG, IGT, HbA1c levels and insulin-sensitivity, the effect on FFA is still controversial. Some studies have reported a decrement in FFA during PEG treatment (78, 84, 85). However in a recent study performed in non-acromegaly patients with type 1 DM, PEG treatment increased hepatic insulin-sensitivity, without significant changes in FFA (86).

Interestingly, one study showed that patients with DM, especially those treated with insulin, require higher PEG doses to normalize IGF-1 levels than those treated with anti-diabetic oral agents (22.8 vs. 17.2 mg pegvisomant/day) (87).

ACROSTUDY is a global surveillance study performed with the aim of monitoring safety and efficacy of PEG (88, 89). Data from the ACROSTUDY Italian register confirmed the overall positive impact of PEG on glucose metabolism. Study population has been divided in two groups, with patients enrolled by centers treating with PEG more than 15 cases belonging to group A and the others to group B. Mean glycaemic levels during PEG treatment decreased in both groups but significantly only in group A (90). Data from the Spanish cohort of ACROSTUDY showed that in diabetic acromegaly patients PEG treatment was able to induce a significant decrease in FPG levels. In line with previous findings, diabetic patients needed higher PEG doses to achieve the biochemical control of acromegaly (91).

In a recent study, 15 patients with well-controlled acromegaly by means of combination therapy with first-generation SSAs plus PEG were switched to PEG therapy only. After 12 months of PEG treatment IGF-1 levels remained controlled in 73% of patients, HbA1c levels decreased significantly, and no change in anti-diabetic medicaments was required (92).

Considering the overall positive impact on glucose homeostasis, PEG treatment represent a good option in patients with uncontrolled diabetes mellitus and resistant to first-generation SSAs (93).

### Dopamine agonists

Bromocriptine and cabergoline are dopamine agonists, which, by binding D2 receptor in the pituitary tumor, can suppress GH secretion. They can be used for the treatment of acromegaly as monotherapy or, more frequently, in combination with SSAs. Bromocriptine can exert a positive impact on glucose metabolism via a direct effect on central nervous system and reducing gluconeogenesis (94, 95). A study performed in 12 acromegaly patients receiving long-term bromocriptine treatment showed a significant decrement in basal and glucose-stimulated insulin levels (96). Cabergoline has been showed to be more effective in the control of acromegaly, compared to bromocriptine (48). Treatment with cabergoline has been associated with weight loss in hyperprolactinaemic patients (97). However, in a pilot study in non-diabetic obese adults, improvement of the glucose tolerance during cabergoline administration was not correlated to weight loss (98). There is a lack of data regarding the impact of dopamine agonists on glucose metabolism in acromegaly. In a multicenter prospective study, the combination therapy with cabergoline and PEG was shown to be more effective in reducing IGF-1 levels than cabergoline or PEG alone, without significant differences in terms of glucose metabolism (99).

## Glucose metabolism alterations and hypopituitarism

Despite the control of GH hypersecretion significantly improves the metabolic complications of acromegaly, DM maintains a higher prevalence in acromegaly patients in remission compared to general population, therefore the follow-up of glucose disorders needs to be performed on a long-term basis also in patients with controlled disease (100).

In addition, metabolic alterations, including varying degrees of glucose metabolism imbalance, may be also the result of hypopituitarism, which can be caused by the GH-secreting pituitary tumor itself or its medical or surgical treatment. Indeed, it is known that untreated GH deficiency (101), hypothyroidism and hypogonadism (102, 103), as well as an overtreatment of adrenal insufficiency, are associated with insulin resistance and may predispose to the development of DM (104). Therefore, a careful management of these patients aiming to control GH hypersecretion and to restore normal endocrine function is mandatory, in order to improve metabolic alterations and to consequently reduce the cardiovascular risk.

## Treatment of DM

There are no specific recommendations for the treatment of acromegaly-associated diabetes. All antidiabetic drugs can be used in acromegaly patients following the stepwise approach generally reserved to type 2 DM subjects (105).

## Author contributions

FF, AA, and AC reviewed the literature and wrote the manuscript; CG and SC critically revised the manuscript.

### Conflict of interest statement

The authors declare that the research was conducted in the absence of any commercial or financial relationships that could be construed as a potential conflict of interest.
